# Retinal Vessel Diameters and Physical Activity in Patients With Mild to Moderate Rheumatic Disease Without Cardiovascular Comorbidities

**DOI:** 10.3389/fphys.2018.00176

**Published:** 2018-03-09

**Authors:** Arne Deiseroth, Thimo Marcin, Colette Berger, Denis Infanger, Juliane Schäfer, Bettina Bannert, Arno Schmidt-Trucksäss, Reinhard E. Voll, Diego Kyburz, Henner Hanssen

**Affiliations:** ^1^Department of Sport, Exercise and Health, University of Basel, Basel, Switzerland; ^2^Rheumatology Unit, University Hospital Basel, Basel, Switzerland; ^3^Department of Rheumatology and Clinical Immunology, University Medical Center Freiburg, Villingen-Schwenningen, Germany

**Keywords:** rheumatic disease, cardiovascular risk, retinal vessel diameter, arterial stiffness, physical activity

## Abstract

**Objectives:** Low-grade systemic inflammation is responsible for atherosclerotic lesions in patients with rheumatic diseases. Vascular dysfunction is a precursor of atherosclerosis and can be improved by physical activity (PA). Our aim was to asses micro- and macrovascular function as well as PA and cardiorespiratory fitness (CRF) in patients with rheumatic diseases in the absence of cardiovascular (CV) comorbidities compared to controls.

**Methods:** Fifty-one patients without CV comorbidities were compared to 35 controls. Retinal microvascular diameters were assessed using a Retinal Vessel Analyzer. Arterial stiffness (AST) was measured by applanation tonometry. CRF was assessed as peak oxygen consumption and PA was assessed with a questionnaire.

**Results:** Retinal venular diameters were significantly wider in patients [median 221 μm (interquartile range (IQR) 211, 231)] compared to controls [median 215 μm (IQR 196, 223); *p* = 0.01]. One hour increase of PA per week led to a venular constriction of −0.56 μm (95%CI −1.09, −0.03; *p* = 0.04). In our patients with low disease activity (median DAS28 1.9; median BASDAI 2.8), no differences in AST were evident compared to controls. The association of PA and CRF with AST was not independent of blood pressure.

**Conclusions:** Patients with rheumatic disease and mild-to-moderate disease activity show an impairment of the retinal microvasculature but not of large artery stiffness. Retinal vessel analysis seems to be a sensitive biomarker to unmask vascular impairments even in the absence of classic CV risk factors. PA may have the potential to counteract the development of small artery disease at early stages of rheumatic disease.

## Introduction

Patients with rheumatic diseases have an increased risk of developing cardiovascular (CV) disease compared to the general population (Hollan et al., 2013). Meta-analyses of observational studies estimate a 50–60% higher mortality of CV diseases in patients with rheumatoid arthritis (RA) (Aviña-Zubieta et al., 2008; Meune et al., 2009). Similar evidence exists for an increased risk of CV disease and CV events in patients with spondyloarthritis (Han et al., 2006; Haroon et al., 2015; Li et al., 2015). High prevalence of CV risk factors in patients with rheumatic diseases may partially, but not fully explain the elevated CV risk (Del Rincón et al., 2001). Atherosclerotic lesions are the main determinants of premature CV deaths in RA and, therefore, keeping an eye on vascular function is of utmost importance in the development and manifestation of rheumatic diseases (Agca et al., 2016).

Arterial stiffness (AST) is a validated vascular biomarker of the macrocirculation and it is frequently used for CV risk stratification (Vlachopoulos et al., 2015). Indices of AST such as pulse wave velocity (PWV), augmentation index (Aix) and central pulse pressure (cPP) have been proven to be strong predictors of future CV events and all-cause mortality in the general population (Vlachopoulos et al., 2010a,b). A number of studies have verified the increased CV risk in patients with severe rheumatic disease (Mäki-Petäjä et al., 2006; Avalos et al., 2007; Bodnár et al., 2011; Costa et al., 2012; Ambrosino et al., 2015).

Microvascular dysfunction has also been shown to be common in rheumatic diseases (Galarraga et al., 2008). Retinal vessel analysis is an emerging technique for the non-invasive assessment of the microvasculature. It allows for the analysis of microvascular structure and function at subclinical stages of CV diseases (Tedeschi-Reiner et al., 2005; Kwa, 2006). Narrow retinal arteries, wider venules and a lower arteriolar to venular ratio (AVR) reflect a higher CV risk in general population (Wong et al., 2002, 2003; Wang et al., 2003, 2007; Seidelmann et al., 2016). In a recent cohort study retinal calibers were independently associated with a higher long-term risk of atherosclerotic CV events (Seidelmann et al., 2016). In the same study, there was a 21% reclassification rate of women with low to intermediate risk. To date, only few studies have investigated and shown alterations of retinal vessels in rheumatic diseases (Van Doornum et al., 2011; Okada et al., 2013).

Systemic inflammation plays a key role for both the development of rheumatic and CV diseases. Chronic inflammation has been argued to be a mediator for the progression of atherosclerosis in patients with rheumatic diseases (Mason and Libby, 2015; Nurmohamed et al., 2015). Inflammation promotes atherosclerosis directly by impairing the vessel wall as well as indirectly by affecting traditional risk factors (Libby, 2012; Skeoch and Bruce, 2015). Inflammation plays a key role in the development of arterial stiffening and anti-inflammatory therapy has been shown to reduce AST and CV risk (Mäki-Petäjä and Wilkinson, 2010). Moreover, anti-inflammatory therapies have also shown to reduce indices retinal vessel diameters (Mäki-Petäjä et al., 2006; Moi et al., 2016).

Exercise is known for its anti-inflammatory properties in the general population (Beavers et al., 2010). High physical activity (PA) and an increase of cardiorespiratory fitness (CRF) have numerous physiological benefits on the CV system, including a reduction in AST (Tanaka and Safar, 2005; Huang et al., 2016). In addition, we have previously shown the positive effects of endurance exercise training on retinal vessel diameters associated with an increase in CRF (Hanssen et al., 2011). Though regular PA is recommended for the management of rheumatic diseases, knowledge about the positive effects of exercise and adherence to guidelines in RA is poor (Tierney et al., 2012; Boo et al., 2017). Moreover, there is only little data on the interplay between CV risk, inflammatory joint diseases and PA in patients with rheumatic diseases (American College of Rheumatology, 2002; Metsios et al., 2008, 2010). Recently, exercise training has proven to ameliorate CV-Risk profile and endothelial function in patients with RA (Stavropoulos-Kalinoglou et al., 2013; Metsios et al., 2014). The associations of PA and retinal vessel diameters in patients with rheumatic diseases are unknown to date.

The aims of the study were twofold. First, we wanted to assess micro- and macro-vascular function of patients with rheumatic diseases in the absence of CV risk factors compared to healthy individuals. Secondly, we aimed to investigate the association of objectively measured CRF and self-reported PA with indices of AST and retinal vessel diameters in patients with mild-to-moderate rheumatic disease.

## Methods

### Study design and subjects

In our cross-sectional study, consecutive patients with rheumatic diseases were recruited from a network of regional hospitals. Either RA, ankylosing spondylitis, psoriatic arthritis, peripheral or axial spondyloarthritis had to be diagnosed at least one year prior to study examination. Controls had to be free of rheumatic diseases and were recruited by advertisement in the university's online marketplace as well as flyer distribution.

Exclusion criteria were history of CV, pulmonary or chronic inflammatory diseases other than of rheumatic origin. Exclusion criteria included CV risk factors such as obesity, defined as body mass index (BMI) ≥ 30 kg/m^2^, and current smoking or smoke cessation less than 10 years ago. Exclusion criteria were assessed through a medical record review and subjects' interviews. Exclusion criteria were identical for patients and controls.

Each participant provided written informed consent and the study was approved by the local ethics committee of Northwest and Central Switzerland (EKNZ2015/235). The study was performed in accordance with the Helsinki Declaration of Good Clinical Practice (World Medical Association, 2013).

### Medical history of patients

Rheumatic diagnosis and medication were collected from medical records obtained from the collaborating hospitals. In addition, all patients filled out a questionnaire to complete the medical information. Disease activity was assessed using the Disease Activity Score 28 (DAS28) for patients with RA and the Bath Ankylosing Spondylitis Disease Activity Index (BASDAI) for patients with ankylosing spondylitis. Patients with psoriatic arthritis or undifferentiated spondyloarthritis were assessed either with the BASDAI in case of axial arthritis, or the DAS28 in case of peripheral arthritis.

The DAS28 was obtained from the hospital, whereas the BASDAI was inquired by the investigators. CRP-levels were obtained from the hospital's database. According to the “American College of Rheumatology” and the “European League Against Rheumatology,” a DAS28 < 2.6 represents minimal disease activity (Felson et al., 2011). A BASDAI value ≥4 is used to define active disease, according to the “Assessment in SpondyloArthritis International Society” (van der Heijde et al., 2011).

### Measurements

#### Preparations and anthropometry

Patients were required to avoid food intake for 3 h and caffeine or alcohol intake for 12 h prior to testing. In addition, all participants were instructed to refrain from exercise 24 h before the examination.Body height and body weight were measured to calculate the BMI (kg/m^2^). Body weight was measured with a body composition analyser (InBody 720 from Biospace co., Ltd., Seoul, Korea).

#### Arterial stiffness

PWV and central hemodynamics were measured as previously described (Townsend et al., 2015). Briefly, AST parameters were assessed non-invasively by applanation tonometry using the SphygmoCor® system (AtCor medical, Sydney, Australia) according to the manufacturer's recommendations (AtCor Medical Pty Ltd., 2010). After resting in supine position, the blood pressure was taken twice with a cuff from the right brachial artery, using an automatic blood pressure monitor system (Omron Healthcare, Mannheim, Germany). Mean value was used for further calculations. Pulse wave velocity (PWV) in m/s was calculated as distance divided by transit time of carotid and femoral pulse wave. The subtraction method was used to measure the distance. The good intra- and inter-observer reproducibility of this technique has been demonstrated in healthy populations and in patients with chronic kidney disease (Frimodt-Møller et al., 2006, 2008). Pulse wave analysis was executed at the right radial artery to calculate central pulse pressure (cPP) and augmentation index (Aix) with the device's in-built software. Since the Aix is influenced by heart rate, the standardized Aix calculated for a set heart rate of 75 beats per minute (Aix@75) was used for further calculations (Wilkinson et al., 2000).

#### Retinal vessel diameter

**Figure 1:**
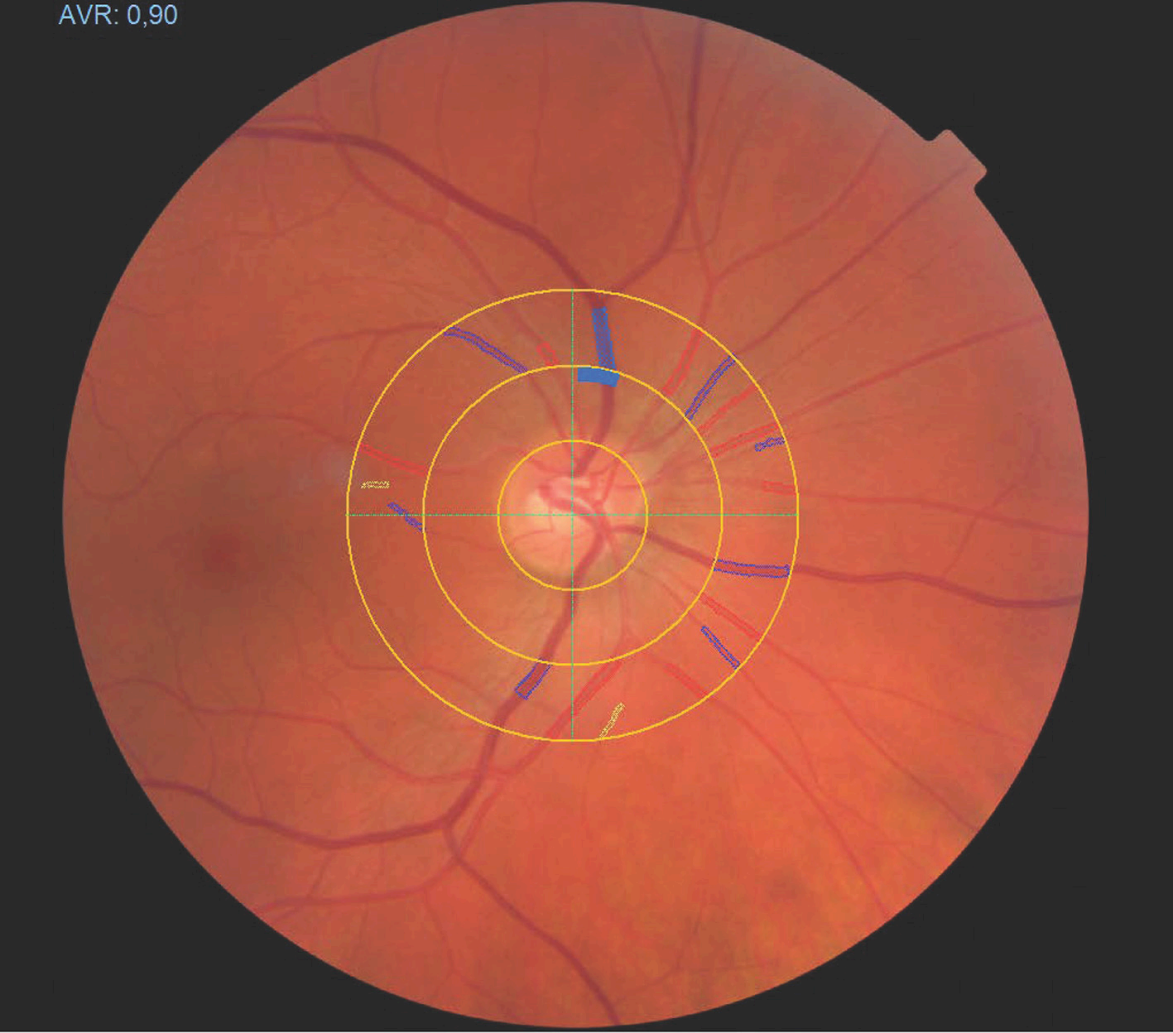
Fundoscopy of the human retina. A software-based identification of retinal vessels in ring-zones allows for measuring of arteriolar and venular diameters.

Central retinal arteriolar equivalent (CRAE), central retinal venular equivalent (CRVE) and arteriovenous ratio (AVR) were measured non-invasively and non-mydriatically using a Static Retinal Vessel Analyzer (SVA-T, Imedos Systems UG, Jena, Germany) (Liew et al., 2008). The system consists of a fundus camera and analyzing software. Two pictures of each eye were taken with the optic disc in the center (Figure 1). Using an analyzing software (Vesselmap 2, Visualis, Imedos Systems UG, Jena, Germany), arterioles and venules, extending over an area of 0.5–1 disc diameters from the origin of the optic nerve, were identified. CRAE and CRVE were calculated by applying the Paar-Hubbard formula (Hubbard et al., 1999). AVR was calculated by dividing CRAE by CRVE. CRAE and CRVE were calculated in measuring units. According to the model of Gullstrand's normal eye, one measuring unit relates to 1 μm. For statistical analysis, mean retinal vessel diameters of both eyes were used. The reproducibility for CRAE and CRVE based in the same fundus photography have been shown to be excellent (Couper et al., 2002). We have previously shown a high reproducibility for the retinal parameters with a correlation coefficient for CRAE of *r* = 0.94 and a coefficient of variation (CoV) of 2.1%. For CRVE, our previously reported correlation coefficient was *r* = 0.95 and the CoV was 2.0% (AVR: *r* = 0.94 and CV = 2.3%) (Imhof et al., 2016).

#### Cardiorespiratory fitness

CRF was assessed by peak oxygen consumption (VO_2_peak), a parameter of maximal aerobic capacity. All participants executed a maximal performance test on a cycle ergometer, following an individual ramp protocol. The slope depended on the predicted maximum of work load, which was estimated by the investigators on the basis of the patient's age, weight and PA derived from the Freiburger Physical Activity Questionnaire (Frey et al., 1999). The individual ramp protocols were set to reach a test duration of 8–12 min as recommended by American College of Sports Medicine (2014). Ventilatory parameters were measured using a calibrated breath-by-breath spiroergometry solution (Metalyzer® 3B, MetaSoft® software from CORTEX Biophysik GmbH, Leipzig, Germany).

#### Physical activity behavior

PA was assessed with a short-form of the self-reported Freiburger Physical Activity Questionnaire. This validated questionnaire allows calculation of total PA (hours per week) as a composite of daily basic activities as well as sport activities (Frey et al., 1999). The questionnaire was modified to investigate inactivity as time spent on television and computer in hours per week.

### Sample size

The primary research hypothesis of this study was that after controlling for age and sex, VO_2_peak will be independently associated with PWV in patients with rheumatic disease. In healthy adults, age and sex have been shown to explain about 25% of the variability in PWV (Vaitkevicius et al., 1993). Given these assumptions, a sample size calculation for a multiple regression determined that 38 patients would have to be included to detect, at 80% power as statistically significant at the 5% level (2-sided), an increase of 15% in the variability attributed to VO_2_peak.

### Statistical analysis

Participant characteristics are presented with median [interquartile range (IQR)] for continuous variables and frequency counts for categorical variables. QQ-Plots and a Kolmogorov-Smirnov-test were used to check whether continuous variables deviated substantially from a normal distribution. To detect statistical differences between patients and controls, a Student's *t*-test was conducted in case of approximately normally distributed data. If variables were notably skewed, Wilcoxon test was applied. χ^2^-test was used for categorical variables. We used multiple linear regression models to estimate the expected change (with 95% confidence interval) in parameters of vascular function per one metabolic equivalent (MET) increase adjusted for age and sex. One MET corresponds to 3.5 ml/kg/min oxygen consumption, which is defined as the amount of oxygen consumed at rest (Jetté et al., 1990). We also used multiple linear regression models to estimate the expected change in vascular function per 1 h per week increase in total PA, sport activity and screen time, adjusted for patient age, sex and BMI (Model 1). In additional analyses, we extended covariate adjustment to include systolic and diastolic blood pressure (Model 2). All statistical tests were two-sided and *P* < 0.05 was considered statistically significant. Data were analyzed using IBM SPSS Statistics for Windows, version 22 (IBM Corp., Armonk, New York, USA).

## Results

### Participant recruitment

**Figure 2:**
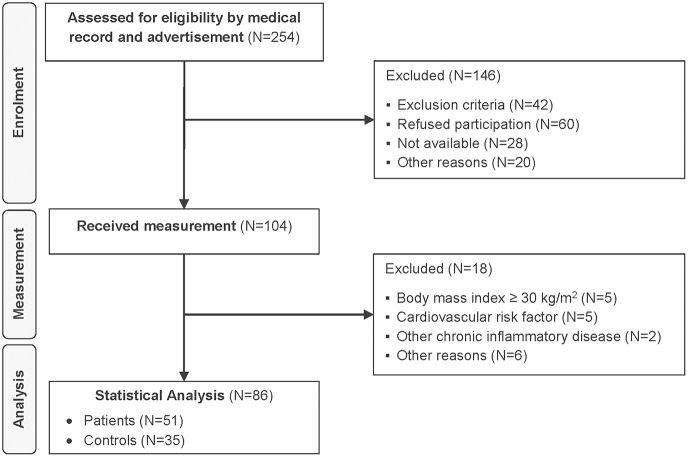
Flow diagram.

A total of 104 patients and healthy controls received measurement (Figure 2). Despite a prior telephone interview, 18 subjects met at least one exclusion criterion. These participants were excluded from analyses to minimize the influence of potentially confounding traditional CV risk factors. In total, 86 participants were included in the statistical analysis, 51 patients and 35 healthy controls.

### Participants' characteristics and group comparison

Sufficient data quality was reached in all patients and allowed statistical inclusion of all retinal images. Due to invalid measurements or unavailable data from the hospitals, not all patient data could be obtained. Participant characteristics as well as group comparisons of patients and controls are shown in Table 1. Both groups included more women. Patients showed higher CRVE levels compared to controls [Median 221 μm (IQR 211, 231) vs. 215 μm (IQR 196, 223); *p* = 0.01]. No statistical difference between patients and controls was found in any of the other vascular indices.

**Table 1:** Participant characteristics.

		**Patients (*****N*** = **51)**			**Controls (*****N*** = **35)**			***p*-value**
		***N***	**Median**	**IQR**	***N***	**Median**	**IQR**	
**BASIC**								
Sex	Female	28			24			0.20
	Male	23			11			
Age [y]		51	51	(42, 57)	35	51	(48, 55)	0.48
Body mass index [kg/m^2^]		51	23.1	(21.2, 26)	35	23.3	(20.8, 25.7)	0.69
pSBP [mmHg]		51	124	(118, 133)	35	125	(119, 133)	0.82
pDBP [mmHg]		51	82	(74, 88)	35	81	(75, 87)	0.88
**VASCULAR FUNCTION**								
CRAE [μm]		51	191	(182, 199)	35	184	(172, 196)	0.06
CRVE [μm]		51	221	(211, 231)	35	215	(196, 223)	0.01
AVR		51	0.86	(0.82, 0.91)	35	0.87	(0.83, 0.92)	0.45
PWV [m/s]		50	7.0	(6.5, 7.7)	34	7.1	(6.4, 8.3)	0.52^*^
Aix [%]		51	23	(13, 31)	35	25	(17, 37)	0.18
Aix@75 [%]		51	16	(5, 27)	35	17	(8, 28)	0.47
cSBP [mmHg]		51	115	(107, 122)	35	114	(110, 126)	0.50
cDBP [mmHg]		51	83	(75, 89)	35	82	(77, 88)	0.93
cPP [mmHg]		51	30	(25, 39)	35	33	(30, 41)	0.09^*^
**PHYSICAL ACTIVITY**								
VO_2_peak [ml/kg/min]	Female	25	31.4	(26.3, 35.6)	22	33.6	(30.3, 40.6)	0.12
	Male	22	41.6	(37.4, 45.7)	11	44.3	(40.8, 52.5)	0.43
Total physical activity [h/week]		51	8.6	(3.9, 12.8)	35	7.3	(4.6, 9.9)	0.49^*^
Sport activities [h/week]		51	1.2	(0.25, 3.8)	35	2.0	(0.75, 3.8)	0.90^*^
Screen time [h/week]		51	35.0	(21, 53)	35	35.0	(14, 53)	0.45

*pSBP, peripheral systolic blood pressure; pDBP, peripheral diastolic blood pressure; CRAE, central retinal arteriolar equivalent; CRVE, central retinal venular equivalent; AVR, arteriovenous ratio; PWV, pulse wave velocity; Aix, augmentation index; Aix@75, standardized augmentation index to a heart rate of 75 beats per minute; cSBP, central systolic blood pressure; cDBP, central diastolic blood pressure; cPP, central pulse pressure; VO_2_peak, peak oxygen consumption; IQR, interquartile range*.

* *Due to lack of normal distribution Wilccoxon test was used*.

Since six participants did not meet exhaustion criteria, they were excluded from further statistical analysis of VO_2_peak. There was no difference regarding CRF or self-reported PA (as assessed by the Freiburger Questionnaire) between the two groups.

Table 2 contains the medical data of the patients. Median disease duration was seven years (IQR 3, 14). Fourty-seven patients were currently treated with at least one anti-rheumatic drug. Most patients exhibited a mild-to-moderate disease activity. Twenty-nine of the patients presented with a minimal disease activity indicated by a DAS28 lower than 2.6 or a BASDAI lower than 4. CRP-concentrations were elevated in four patients only.

**Table 2:** Medical record of patients with rheumatic diseases.

	***N***	**Median**	**IQR**
**Rheumatic disease**	51		
Rheumatoid arthritis	26		
Psoriatic arthritis	13		
Ankylosing spondylitis	6		
Undifferentiated spondyloarthritis	6		
**Antirheumatic drug intake**	47		
NSAID intake	23		
Cortison intake	17		
Methotrexat intake	15		
TNFα-Inhibitor intake	18		
Other DMARD intake	21		
Disease duration [y]	51	7	(3, 14)
Disease Activity Score 28	28	1.9	(1.6, 3.3)
Bath Ankylosing Spondylitis Disease Activity Index	17	2.8	(1.1, 4.4)
CRP [>10 mg/l]	4/49		

*NSAID, nonsteroidal anti-inflammatory drug; TNF, tumor necrosis factor; DMARD, disease-modifying anti-rheumatic drug; CRP, c reactive protein; IQR, interquartile range*.

### Influence of fitness and activity parameters on vascular function

The results of the multiple linear regression model are presented in Tables 3, 4. After controlling for age and sex, CRVE was inversely associated with PA in patients with rheumatic diseases. A rise by 1 h per week in self-reported total PA resulted in a decrease of CRVE of −0.59 μm (IQR −1.10, −0.08) after adjustment for age, sex and BMI (Model 1; *p* = 0.02) (Table 3). This association remained significant when further adjusted for blood pressure [Model 2; β = −0.56 μm (IQR −1.09, −0.03); *p* = 0.04]. Although self-reported sports activity does not seem to impact on CRAE and CRVE separately, AVR increases with higher weekly participation in leisure time sports. Per 1 h increase of sports participation, AVR decreased by 0.005 (Model 2; IQR 0.000, 0.009; *p* = 0.03). VO_2_peak and screen time did not show associations with retinal vessel diameters.

**Table 3:** Influence of fitness and activity parameters on retinal vessels.

	**Model**	**CRAE [μm]**			**CRVE [μm]**			**AVR**		
		***R*^2^**	**Coefficient (95% CI)**	***p*-value**	***R*^2^**	**Coefficient (95% CI)**	***p*-value**	***R*^2^**	**Coefficient (95% CI)**	***p-v*alue**
VO_2_peak^a^ (*N* = 47)	1	0.08	−1.04 (−3.37, 1.30)	0.37	0.06	−0.76 (−3.50, 1.97)	0.58	0.02	−0.003 (−0.013, 0.007)	0.59
	2	0.15	−1.07 (−3.41, 1.26)	0.36	0.19	−0.30 (−2.94, 2.33)	0.82	0.34	−0.005 (−0.013, 0.004)	0.28
Total physical activity^b^ (*N* = 50)	1	0.05	−0.06 (−0.51, 0.38)	0.78	0.14	−0.59 (−1.10, −0.08)	0.02	0.10	0.002 (0.000, 0.004)	0.04
	2	0.14	−0.20 (−0.66, 0.25)	0.37	0.19	−0.56 (−1.09, −0.03)	0.04	0.27	0.001 (−0.001, 0.003)	0.17
Sport activity^b^ (*N* = 50)	1	0.05	0.16 (−1.00, 1.33)	0.78	0.10	−1.19 (−2.54, 0.17)	0.08	0.13	0.006 (0.001, 0.011)	0.02
	2	0.12	0.08 (−1.08, 1.23)	0.89	0.15	−1.04 (−2.40, 0.31)	0.13	0.32	0.005 (0.000, 0.009)	0.03
Screen time^b^ (*N* = 50)	1	0.06	−0.08 (−0.35, 0.18)	0.53	0.05	−0.11 (−0.42, 0.21)	0.51	0.01	0.000 (−0.001, 0.001)	0.95
	2	0.12	−0.03 (−0.31, 0.24)	0.81	0.11	−0.09 (−0.42, 0.24)	0.59	0.24	0.000 (−0.001, 0.001)	0.86

*Central retinal arteriolar equivalent (CRAE), central retinal venular equivalent (CRVE) and arteriovenous ratio (AVR) were used as dependent variables in a multiple linear regression*.

*Model 1, adjusted for age, sex and body mass index; Model 2 , Model 1 additionally adjusted for systolic and diastolic blood pressure*.

*VO_2_peak, peak oxygen consumption; MET, metabolic equivalent; CI, confidence interval*.

a *By 1 MET increase*.

b *By 1 h per week increase*.

**Table 4:** Influence of fitness and activity parameters on arterial stiffness.

	**Model**	**PWV [m/s]**			**Aix@75 [%]**			**cPP [mmHg]**		
		***R*^2^**	**Coefficient (95% CI)**	***p*-value**	**R^2^**	**Coefficient (95% CI)**	***p*-value**	***R*^2^**	**Coefficient (95% CI)**	***p*-value**
VO_2_peak^a^ (*N* = 47)	1	0.26	−0.07 (−0.22, 0.07)	0.33	0.66	−1.17 (−2.34, 0.01)	0.05	0.36	−0.03 (−1.18, 1.12)	0.95
	2	0.35	0.08 (−0.23, 0.06)	0.26	0.70	−1.04 (−2.18, −0.10)	0.07	0.92	−0.38 (−0.80, 0.04)	0.07
Total physical activity^b^ (*N* = 50)	1	0.35	−0.03 (−0.06, −0.00)	0.03	0.65	−0.23 (−0.46, 0.00)	0.05	0.40	−0.17 (−0.40, 0.05)	0.13
	2	0.40	−0.02 (−0.05, 0.00)	0.09	0.68	−0.16 (−0.40, 0.08)	0.17	0.92	−0.05 (−0.14, 0.04)	0.26
Sport activity^b^ (*N* = 50)	1	0.33	−0.07 (−0.14, 0.00)	0.05	0.65	−0.58 (−1.19, 0.03)	0.06	0.36	−0.07 (−0.67, 0.53)	0.81
	2	0.41	−0.07 (−0.14, 0.00)	0.06	0.69	−0.50 (−1.10, 0.09)	0.10	0.92	−0.12 (−0.34, −0.34)	0.27
Screen time^b^ (*N* = 50)	1	0.30	0.01 (0.00, 0.03)	0.16	0.62	0.03 (−0.12, 0.17)	0.69	0.44	0.16 (0.03, 0.29)	0.02
	2	0.37	0.01 (−0.01, 0.02)	0.42	0.67	0.01 (−0.14, 0.16)	0.88	0.92	0.02 (−0.03, 0.07)	0.48

*Pulse wave velocity (PWV), standardized augmentation index to a heart rate of 75 beats per minute (Aix@75) and central pulse pressure (cPP) were used as dependent variables in a multiple linear regression*.

*Model 1, adjusted for age, sex and body mass index; Model 2, Model 1 additionally adjusted for systolic and diastolic blood pressure*.

*VO_2_peak, peak oxygen consumption; MET, metabolic equivalent; CI, confidence interval*.

a *By 1 MET increase*.

b *By 1 h per week increase*.

In our patients higher CRF-levels and self-reported PA were associated with lower indices of AST (Table 4). However, these associations depended on blood pressure and lost significance after its adjustment. For example, increase of self-reported PA by 1 h resulted in a PWV decrease of 0.03 m/s (Model 1; IQR −0.06, 0.00; *p* = 0.03). After further adjustment for blood pressure (Model 2), significance was lost. A similar trend was evident for total PA and Aix@75. Inactivity as measured by screen time did not seem to affect indices of AST.

## Discussion

Our study examined macro- and micro-vascular impairments in patients with mild-to-moderate rheumatic diseases compared to controls. Furthermore, we investigated the influence of CRF and PA on vascular function in these patients. Our results showed wider retinal venular diameters, a microvascular biomarker for increased CV risk, in patients with rheumatic diseases. Higher PA levels were independently associated with retinal venular constriction, indicating therapeutic potential for the microvascular impairment in patients with rheumatic disease. No differences in large artery stiffness were found between the two groups. Significant associations between PA and CRF with PWV and central hemodynamics in patients with rheumatic diseases depended on adjustment for blood pressure.

Previous studies have found significantly higher values of AST in patients with rheumatic diseases. In a meta-analysis, Ambrosino et al. calculated PWV to be 1.3 m/s higher in patients, which was accompanied with an 11.5% higher AIx compared to healthy controls (Ambrosino et al., 2015). We did not find increased AST in our group of patients. The main reasons for this discrepancy lie in the study design and the patients' characteristics. We took great care in recruiting patients without underlying CV risk factors. This also explains our comparatively small sample size. In previous studies, vascular impairments may have been explained by CV comorbidities. Our patients had a mild-to-moderate disease activity and were free of classic CV risk factors. Therefore, we assume that the retinal microvascular impairments of our patients can be traced back to the rheumatic inflammatory disease. Only four out of 49 patients with available data presented with elevated CRP levels. DAS28 and BASDAI were low, indicating sufficient disease control by anti-rheumatic medication (Table 2). Median DAS28, for example, was 1.9 in our patients and varied between 3.0 and 5.8 in the studies included in the meta-analysis by Ambrosino et al. (2015).

Previous studies reported low fitness levels in patients with rheumatic diseases (Metsios et al., 2015; O'Dwyer et al., 2015). In our study patients did not have lower fitness levels compared to their healthy counterparts. Metsios et al. report RA patients to have a mean maximal oxygen consumption of 20.9 ml/kg/min in 150 RA patients (Metsios et al., 2015). In our study, median VO_2_peak was 31 ml/kg/min among female and 42 ml/kg/min among male patients. In comparison to normal age-related values for a general population, this corresponds to the 50th percentile in our female patients and to the 75th percentile in our male patients (American College of Sports Medicine, 2014). Therefore, our patients had a low disease activity, were relatively fit and were free of classical CV risk factors.

Retinal fundoscopy allows for an easy and non-invasive evaluation of the human microcirculation. Our results confirm the findings of a previous study reporting wider retinal venules in patients with rheumatic diseases. Van Doornum et al. were the first to report retinal venular dilatation in 51 RA patients compared to age- and gender-matches controls (Van Doornum et al., 2011). Their results were verified and generalized to a wider spectrum of rheumatic diseases by Okada et al. (2013). The patients of these studies were not free of CV risk and presented with higher disease activity. Patients of both studies reveal a broad spectrum of CV diseases which were associated with retinal venular dilatation (Wang et al., 2006b; Wong et al., 2006). Microvascular impairments may have occurred due to their cardiometabolic comorbidities rather than the rheumatic disease itself.

Recently, Aissopou et al. compared 188 patients with controls free of chronic systemic inflammatory diseases (Aissopou et al., 2017). The authors postulated normal retinal venular diameter in patients with rheumatic diseases and explained their results with a low disease activity due to optimal medical disease control. However, 64% of their controls were hypertensive and thereby not free of CV comorbidities. Our study is the first to show retinal microvascular impairments even in patients with mild-to-moderate disease activity and in the absence of CV comorbidities or risk factors. Our results corroborate the common assumption that chronic systemic inflammation leads to production of cytokines and is the underlying cause for the increased prevalence of CV disease in patients with rheumatic diseases.

Our findings are in line with previous reports for the Beaver Dam Eye Study showing an association of inflammatory markers with wider retinal diameters (Klein et al., 2006). Wider retinal vessel diameters have been associated with an increased risk of stroke (Ikram et al., 2006) and risk for coronary heart disease (Wang et al., 2006a). Retinal venules have shown consistent associations with CV and non-CV mortality in a general population (Mutlu et al., 2016). Both narrower retinal arteries and wider retinal venules can predict risk of death from coronary heart disease and stroke (Wang et al., 2007). In a recent analysis of the Atherosclerosis Risk in Communities study, both arteriolar narrowing and venular widening were associated with atherosclerotic cardiovascular events, coronary heart disease, stroke and death and retinal vessel diameters reclassified 21% of low risk women as intermediate risk (Seidelmann et al., 2016). Based on the predictive value of retinal venular diameters, patients with rheumatic disease without CV comorbidities seem to be at considerably higher risk of CV events even in case of mild-to-moderate disease activity.

How can the adverse risk prediction be altered and what are the effects of anti-inflammatory therapies on the retinal vessels? Recently, Moi et al. showed that pharmacological suppression of inflammation in RA patients was accompanied by a reduction of retinal venular diameter (Moi et al., 2016). Regular exercise is also known for its anti-inflammatory properties (Beavers et al., 2010). Whether PA and fitness are associated with improved CV health in patients with rheumatic diseases has not been examined before. We have previously shown that regular endurance exercise can induce arteriolar dilatation and venular constriction in lean and obese adults (Hanssen et al., 2011). Though not of interventional character, the results of our present study suggest that higher levels of daily PA can counteract microvascular impairments in patients with chronic inflammatory diseases. In addition, sports activities were associated with a higher and favorable AVR in our patients. Compared to normal daily activities (e.g., household) sports activities are associated with higher exercise intensities. Future studies will have to investigate whether higher exercise intensities have more pronounced effects on microvascular health in chronic inflammatory disease states.

The results of our study suggest that retinal vessel diameters are a more sensitive vascular biomarker than large artery stiffness in patients with mild-to-moderate rheumatic disease. Our patients showed no impairments of AST and the association with PA and CRF was not independent of blood pressure. Crilly and Wallace examined 114 patients with rheumatic diseases and found lower Aix@75 in patients with higher activity scores (Crilly and Wallace, 2013). In our study, Aix@75 was also associated with PA but did not remain significant after adjustment for blood pressure.

Few limitations of the study need to be addressed. This is a cross-sectional study and cannot claim for causality. Furthermore, self-reported PA in our study was assessed with the help of questionnaires, which have their limitations (Haskell, 2012). The study was powered to detect an effect of CRF (VO_2_peak) on large artery stiffness (PWV) in patients with rheumatic disease after controlling for age and sex. Therefore, results regarding retinal vessel diameters are in part associated with a relatively large variability due to insufficient power. However, our sample size resembles that of other studies in the field. We attracted fit and physically active patients, whereas patients with a higher disease activity might have been discouraged by the maximal fitness test protocol. Three different entities of rheumatic diseases were included in our study. Since we were eager to recruit patients without cardiovascular diseases, recruitment of a single rheumatic disease was not possible. However, systemic inflammation and its effects on the vasculature are similar in the three entities. Moreover, female patients and controls outnumber men in our study. Yet this represents the normal sex distribution in rheumatic diseases (Scott et al., 2010). Patients in our study were under a broad spectrum of anti-inflammatory medication, which may have affected the vasculature. This can be considered representative of the medication in daily routine.

The results of our study have important clinical implications. The beneficial effects of exercise seem manifold. The anti-inflammatory effects of PA may not only improve disease-related immunological imbalances, but may also ameliorate atherosclerotic disease development in patients with rheumatic diseases. Whether or not this manifests in a long-term reduction of CV mortality would have to be investigated in future prospective exercise intervention trials.

In conclusion, patients with rheumatic diseases and mild-to-moderate disease activity showed a microvascular but not large artery impairment compared to healthy controls. This was evident even in the absence of CV comorbidities or classical risk factors. PA was inversely associated with favorable narrower retinal venules and may prove to be a treatment option to reduce CV risk in patients with early stage rheumatic diseases. Retinal vessel imaging seems to be a valid diagnostic tool to monitor CV disease development in patients with rheumatic disease. Longer-term prospective outcome studies are necessary to evaluate the prognostic value of retinal vessel diameters in rheumatic disease.

## Ethics statement

All subjects gave written informed consent in accordance with the Declaration of Helsinki. The protocol was approved by the Ethikkommission Nordwest- und Zentralschweiz (EKNZ).

## Author contributions

AD, TM, CB, JS, AS-T, and HH: Were responsible for conception and design of the study; AD, TM, CB, BB, RV, DK, and HH: Were involved in recruitment of patients; AD, TM, CB, and HH: Performed the measurements; AD, TM, CB, DI, JS, and HH: Planned and performed the statistical analysis; AD wrote the first draft of the manuscript. All authors contributed substantially to manuscript revision, read and approved the submitted version.

### Conflict of interest statement

JS has been an employee of F. Hoffmann-La Roche Ltd since December 1, 2016. The present study was conducted before JS joined F. Hoffmann-La Roche Ltd and has no connection to her employment by the company. The other authors declare that the research was conducted in the absence of any commercial or financial relationships that could be construed as a potential conflict of interest.
